# Implementation of the Healthy Workplace Participatory Program in a Retail Setting: A Feasibility Study and Framework for Evaluation

**DOI:** 10.3390/ijerph16040590

**Published:** 2019-02-18

**Authors:** Jaime R. Strickland, Anna M. Kinghorn, Bradley A. Evanoff, Ann Marie Dale

**Affiliations:** Division of General Medical Sciences, Washington University School of Medicine, Saint Louis, MO 63110, USA; akinghorn@wustl.edu (A.M.K.); bevanoff@wustl.edu (B.A.E.); amdale@wustl.edu (A.M.D.)

**Keywords:** Total Worker Health, participatory methods, program implementation, organizational readiness, process evaluation, logic model

## Abstract

Participatory methods used in Total Worker Health^®^ programs have not been well studied, and little is known about what is needed to successfully implement these programs. We conducted a participatory health promotion program with grocery store workers using the Healthy Workplace Participatory Program (HWPP) from the Center for the Promotion of Health in the New England Workplace. We recruited a design team made up of six line-level workers and a steering committee with management and union representatives; a research team member facilitated the program. Using a formal evaluation framework, we measured program implementation including workplace context, fidelity to HWPP materials, design team and steering committee engagement, program outputs, and perceptions of the program. The HWPP was moderately successful in this setting, but required a substantial amount of worker and facilitator time. Design team members did not have the skills needed to move through the process and the steering committee did not offer adequate support to compensate for the team’s shortfall. The evaluation framework provided a simple and practical method for identifying barriers to program delivery. Future studies should address these barriers to delivery and explore translation of this program to other settings.

## 1. Introduction

More than one-third of current U.S. workers suffer from at least one chronic disease, including heart disease, cancer, diabetes, stroke, and musculoskeletal disorders [1,2]. Working adults with chronic disease are more likely to have a reduced working capacity and greater difficulty staying at work than their healthy peers [3,4]. These chronic health conditions have an enormous impact in the lives of workers, but they also place a burden on their employers [3,5]. Healthy behaviors can reduce the effects of chronic conditions for better work (fewer missed days, increased productivity) and health (less musculoskeletal pain, improved mental health) outcomes [5,6,7,8,9,10].

The workplace is an ideal place for supporting healthy behaviors, since workers spend a large portion of their day in the work environment and coworkers and supervisors can provide substantial support. Traditionally, worksite health promotion programs have been separate from other occupational health and safety efforts, and usually target only the individual, ignoring work organization and work environment factors that affect worker behavior. The National Institute for Occupational Safety and Health (NIOSH’s) Total Worker Health^®^ (TWH) approach highlights the need for “policies, programs, and practices that integrate protection from work-related safety and health hazards with promotion of injury and illness prevention efforts to advance worker well-being” [11]. The TWH approach recognizes that work is a social determinant of health, and that workplace factors such as work hours, relationships with coworkers and supervisors, and access to health and wellness programs have important effects on worker health and well-being. Further, TWH principles recognize the Hierarchy of Controls framework to illustrate that system-level interventions are more effective than individual-level interventions [12].

Regardless of the level of intervention, the most effective interventions are those that take into consideration the unique characteristics and perspectives of the end users [13,14]. Participatory methods such as Participatory Action Research and Participatory Ergonomics promote the inclusion of end users in the intervention development process [14,15,16,17,18,19,20]. These end users may be line-level workers who directly benefit from the intervention, managers or others who implement and monitor interventions, or others who are impacted by the interventions in some way. Including these users in the process allows their perspectives to be considered in identifying both workplace health hazards and possible barriers to adopting or participating in the planned interventions. Participatory methods are increasingly being used in Total Worker Health research and practice [14,17,21,22,23,24,25,26,27,28]. The most thoroughly studied participatory program in the TWH literature to date is the Healthy Workplace Participatory Program (HWPP) developed by the Center for the Promotion of Health in the New England Workplace (CPH-NEW). The HWPP is a worker-management participatory program designed to develop solutions for workplace problems that involve front-line workers. The freely available online program includes step-by-step guidance for assembling the participants, identifying problems, and developing and implementing solutions. The developers note the importance of organizational readiness and leadership support, and have recently developed a checklist to measure organizational readiness as well as a Process Evaluation Rating Sheet (PERS) and Management Dashboard [18,29]. This promising and relatively new program has been used in various work settings including corrections facilities, real estate, non-profit healthcare and social assistance agencies, and state government executive offices [28,30]. Publications to date provide little practical advice for implementing the HWPP program (e.g., characteristics most important for success, total time commitment, expectations of the design team, facilitator role). Further, the TWH literature as a whole discusses the utility of participatory approaches, but offers little guidance on how to comprehensively evaluate both implementation and efficacy of these programs while simultaneously considering the contexts in which they are delivered [12,13,31,32].

We sought to evaluate the feasibility of conducting a participatory health promotion program in a retail grocery store setting. We partnered with a regional grocery store chain who expressed interest in supporting their workers’ health. Using the HWPP as a facilitation guide, we formed a team of grocery store workers and evaluated their ability to create meaningful and relevant workplace health activities that promote and support healthy behaviors in their workforce. The purpose of this paper is twofold: (1) to inform others considering a participatory intervention by describing the implementation of this HWPP program, and (2) to describe a framework for evaluating complex TWH interventions, such as the HWPP.

## 2. Materials and Methods

### 2.1. Overview and Employer Context

This study was an extension of a partnership with a labor union and several regional grocery store chains who had participated in a preliminary study examining workplace factors related to health behaviors and obesity [33,34]. Upon completion of that study, we approached our partners about piloting the HWPP in one store. We explained that the goal of the program was to develop and implement health and wellness initiatives to promote health in the workplace setting and support workers’ efforts to make positive health changes; one of the grocers agreed to participate.

The study period was from September 2014 to June 2016, during which time we piloted the HWPP program, collected process measures, and collected baseline and follow-up worker assessments by surveys and focus groups. The Institutional Review Board at Washington University approved all research activities and all participants provided informed consent.

### 2.2. Program Description

#### 2.2.1. HWPP Model and IDEAS Tool

The HWPP model includes a design team made up of front-line workers and a steering committee comprised of multiple management levels [35]. These two teams work together, with the help of a program facilitator, to create health and wellness activities for their workplace. The model uses the Intervention Design and Analysis Scorecard (IDEAS Tool) which includes seven steps: (1) identify problems and contributing factors, (2) develop intervention objectives and activities, (3) set selection criteria, (4) apply selection criteria, (5A) rate intervention activities, (5B) select intervention activities, (6) plan and implement intervention activities, and 7) monitor and evaluate intervention activities [21,36]. With the guidance of the facilitator, the design team works through these steps using worksheets to create intervention options (Steps 1–5A) to present to the steering committee (Step 5B); both teams work together to implement and monitor the intervention activities (Steps 6–7).

#### 2.2.2. Planning & Roles

At study initiation, the research team met with the grocer’s management to describe the study and outline the project’s goal: To trial a participatory process as a method to generate ideas that promote worker health. They outlined the rationale for participatory programs and discussed the expectations and roles of both the employer (i.e., grocer) and research team. The grocer was willing to trial the program in one store and agreed to: (1) help form a representative steering committee and design team; (2) assist with scheduling design team meetings and allowing design team members to meet during work hours, provided they clock out for meetings; (3) provide a meeting space; and (4) provide access to store workers for data collection. It was expected that the research team would assume responsibility and costs for program facilitation and data collection. The research team also made the decision to pay design team members for their time to attend meetings since they were not able to meet on paid work time; they were paid $25 per meeting.

A research team member with experience in workplace interventions and group facilitation served as the facilitator; two additional research team members assisted in program development and attended meetings to collect process measures. The facilitator’s role was to guide the Design Team through the IDEAS Tool by teaching them the process, planning and running team meetings, and acting as a liaison between the Design Team & Steering Committee. Along with the research team, the facilitator created an agenda and timeline based on the IDEAS Tool and activities from the HWPP toolkit [36]. The initial program plan consisted of seven, one-hour meetings over the course of nine weeks, with two optional meetings scheduled if needed to complete steps 1–5A of the IDEAS Tool. Considering that the program was initiated within the context of a time-limited research study, the facilitator’s goal was to complete one or two cycles of the IDEAS Tool with the Design Team and identify a leader from among the group who could assume the facilitator role and thus ensure program sustainability beyond the study period. Additionally, the HWPP model suggests that employers collect baseline data on the workforce characteristics and health status, and environment or work processes that would aid the design team to creating meaningful interventions [35]. The research team took responsibility for collecting this data; we conducted worker surveys (*n* = 120) and focus groups (*n* = 19) to gather information about current health status, behaviors, and health beliefs of store workers, as well as information about existing workplace supports for health [37,38,39,40,41,42,43,44]. The Design Team’s main role was to complete the IDEAS Tool worksheets, creating intervention options relevant to their work environment to present to the Steering Committee for consideration. After Steering Committee approval, the Design Team was to work together with the Steering Committee to finalize and implement intervention activities. While the majority of the program was designed to take place during team meetings, design team members were expected to complete ‘homework’ tasks between meetings in order to increase productivity during meeting time; these homework tasks were to take approximately 30–60 min to complete each week.

#### 2.2.3. Experience Map

**Figure 1:**
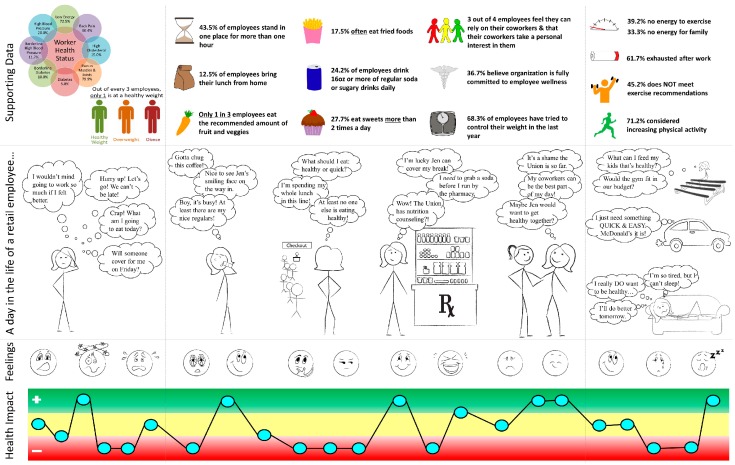
Experience Map.

The research team used experience mapping as way to present the baseline data to the design team in a simple and meaningful way. To complement the survey and focus group data already collected, design team members were asked to complete a store mapping activity in which they drew their store layout and mapped their routes throughout the workday, noting their perceptions of the positive, neutral, and negative impacts on their health. The totality of the formative research was synthesized by the research team and used to create an experience map (Figure 1) that was presented to the design team to use throughout the program [45].

The experience map’s central focus was a persona describing “a day in the life of a grocery store employee.” This story included both work and non-work time to highlight the importance of examining both workplace and personal factors to understand health behaviors and outcomes. Also included in the map was quantitative data from the surveys as supporting evidence for the persona, including disease and symptom rates (e.g., obesity, diabetes, back pain), information about current health behaviors (e.g., diet and exercise), and perceptions of workplace influences on health and organizational commitment to employee health. We used a variety of graphics and images to convey ideas and emotions that are not easily expressed in words and numbers. This enabled the research team to present complex information back to the design team in simple graphic format which they could easily digest and utilize to efficiently identify health priorities and goals, workplace barriers to health, and opportunities for intervention.

#### 2.2.4. Design Team and Steering Committee Recruitment

Store workers volunteered for the design team at the time they completed their baseline survey. Because the program is largely driven by the design team, it was essential that we included workers who were interested in the topic and therefore more likely to remain engaged throughout the process. We used the selection criteria outlined in the HWPP Toolkit as a guide for identifying and selecting six to eight workers with the help of store management [46]. The HWPP suggested that team members should (1) represent all line-level jobs and task environments, (2) represent the demographics of line-level workers, (3) be committed to health and safety and/or improving the workplace, (4) be willing to work together, (5) be open to learning new skills, (6) be able to function as an opinion leader, and (7) be able to meet on a regular basis (missing no more than two meetings). The HWPP also provided guidance on selection of the Steering Committee indicating that they should (1) occupy different levels and roles within the organization, (2) be knowledgeable, or interested, in the area of health promotion/protection, (3) have authority to authorize programs and funding as needed, (4) represent and have the respect of a large number of the workforce, (5) be able to coordinate activities of the Healthy Workplace Project with standing committees. When we formed the steering committee [47], we sought approval and participation from the two larger union locals because eligible, unionized workers received health benefits through their union; a representative from these locals agreed to participate. The steering committee also included the storefront supervisor (as a proxy for the store manager), a representative from corporate labor relations, and a representative from corporate human resources. We did not include representatives from the other unions due to the small number of workers they represented.

### 2.3. Process Evaluation

#### 2.3.1. Logic Model

**Figure 2:**
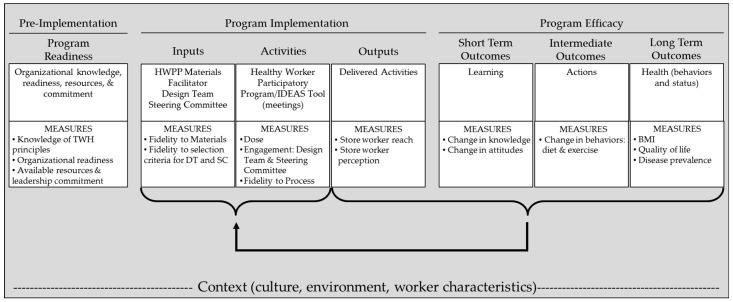
Logic model for evaluating the Healthy Workplace Participatory Program (HWPP).

We created a logic model to guide our evaluation of the HWPP implementation process (Figure 2). We adapted this model from our previously published work in participatory ergonomics [48,49] and incorporated elements that are common in program evaluation [50,51,52].

The logic model begins at the left with “Pre-Implementation” elements (i.e., organizational knowledge, readiness, dedicated resources, and leadership commitment) in order to assess the preparedness of the workplace to initiate a TWH program. This allows the researcher to provide the necessary training and education on TWH before the program begins, creating the foundation for program implementation. The next section shows elements related to the program implementation process, including the inputs (i.e., the resources put into the project), activities (i.e., what the program entails), and outputs (i.e., what was accomplished). The right side of the model shows efficacy measures including the short-term, intermediate, and long-term outcomes the program is indented to produce. All elements are imbedded within the organizational context, which may directly or indirectly influence program or intervention success. Although the model may coincide with time, it is not intended to be a linear evaluation, but rather a continuous, iterative process. As indicated by the brackets, the evaluation of outputs and outcomes will be fed back to inform inputs and activities. This circular process allows periodic evaluation and adjustment of the program as necessary. The evaluation in this paper focuses specifically on the implementation process within the context of a unionized grocery store setting. Due to time and resource limitations, we did not measure pre-implementation elements or program efficacy.

#### 2.3.2. Data Collection

To measure program implementation, the research team collected both qualitative and quantitative data using multiple tools. For each design team meeting, the research team completed field logs and debriefing notes to measure dose (frequency and duration of meetings) and fidelity to the HWPP materials and IDEAS Tool, and rated four dimensions of team member engagement (offered new ideas during meetings, actively participated in meeting, completed homework, and discussed projects with co-workers) on a 3-point scale (0 = no; 0.5 = somewhat; 1 = yes). The design team also rated their own participation and completed meeting reflections [53]. After the completion of the program, the design team members completed semi-structured interviews and a short survey to record final perceptions of both the program and the team’s ability to move forward with implementing solutions without the support of the research team. All store workers were surveyed about their awareness and utilization of the implemented activities three months after they were implemented. The survey asked what changes related to health and wellness they had noticed in their store over the study period, and if they had participated in any of the health activities. We asked if any of the activities “helped them improve (their) eating/and or exercise habits,” what limitations prevented them from participating in the health activities listed, and if the activities were relevant to their life. We also conducted semi-structured interviews with five store workers to further gauge their perceptions of the activities implemented in their workplace.

#### 2.3.3. Data Analysis

We used SPSS v. 23 (IBM, Armonk, NY, USA) to run descriptive statistics for baseline demographics and with store worker follow up surveys for program reach metrics (i.e., awareness and use of activities). We rated all process components according to the measures described in our logic model. A process measure of design team participation was the average rating of each team member’s engagement scores across meetings. Qualitative data was not systematically coded, but each qualitative item was reviewed with consensus by the research team to summarize each process measure. Qualitative data was also used to provide descriptive information to support the quantitative results.

## 3. Results

### 3.1. Model Context

The participating grocery store chain offered a large, busy store that was located in a demographically diverse neighborhood. This store was chosen because of the diverse employee and customer demographics, as well as the store manager, who was enthusiastic about the program. After the initiation of the program, this store manager was transferred to another store; the replacement manager was not as invested in the study. During the project planning phase, store management agreed to adjust work schedules of design team members so they would be scheduled to work on meeting days, and could attend meetings immediately before or after their scheduled shift; this did not always happen over the course of the program. Store management provided a space for the design team to meet on site, although it was not always private due to limited space options in the store.

The selected store employed approximately 159 workers, roughly 40% of whom were full-time employees. We obtained baseline surveys from 120 workers (75% response rate); their demographics are presented in Table 1. The majority of the workforce was unionized and represented by one of five different unions/locals within the store.

### 3.2. Model Inputs

We used all of the IDEAS Tool worksheets, but simplified some of the language to make them more understandable to the design team members. The design team reported that although they understood the program materials when the facilitator guided the process, the worksheets were not intuitive to complete on their own. Thus, the facilitator was a critical part of the team’s success in completing the steps of the IDEAS Tool. The facilitator devoted considerable time over the course of the program to prep, plan, and facilitate team meetings. The majority of the facilitator’s time was spent between meetings, combing through the design team’s materials to condense and simplify the information to help move the team through the program (Table 2). The criteria for recruitment for the design team and steering committee were met. However, store management was not able to consistently schedule team members to work on the day of the meeting as planned so not all team members were able to attend the weekly meetings. Seven workers were initially recruited, but one was unable to regularly attend the meetings. The final design team consisted of six workers with racial and gender diversity. The team was representative of the line-level workers in terms of age, seniority, union membership, and self-reported weight. We recruited a volunteer from six of the store’s largest departments. The six departments with design team volunteers represented 52% of the store’s workforce.

### 3.3. Model Activities

Fidelity to the IDEAS process was met and Steps 1–6 were completed by the design team or steering committee (Table 2). Step 7 (evaluation) was not completed by the design team or steering committee, as formative and follow-up survey data collection was completed by the research team. Design team members were highly engaged during the meetings and attendance was consistent; no design team member missed more than two meetings and they often attended meetings on their days off work. While the level of participation during meetings varied by person, all team members contributed to the discussion and offered new ideas. The members of the design team were not consistent with completing assigned ‘homework’ tasks outside of meetings, but they did report talking to each other about the program between meetings. Scheduling conflicts and other priorities prevented greater time for discussion and completion of homework activities.

Overall, design team members had positive perceptions of the program. They reported that the program met their expectations and positively influenced their health (i.e., drinking more water, purchasing healthier food options). Five of the six team members felt that the participatory process created opportunities for more open dialogue with management, although they did not feel confident that management would follow-through on implementing proposed activities. In addition, a few design team members were frustrated with being scheduled to work at the time of the team meetings, however they were able to work with their immediate supervisor to attend. Early in the process (Step 1), the design team participated in two rounds of brainstorming which generated a total of 65 ideas grouped into four themes (diet, physical activity, stress, and health awareness). The team referred to these ideas and themes in Step 2 to identify their goal (“Reduce Stress at Work”), develop three objectives (“Improve Diet at Work,” “Improve Store Communication,” and “Increase Health Awareness”), and create 15 specific activities related to the three objectives. The design team rated these activities during Steps 3 and 4 with the understanding that they would have to “sell” the ideas to management. The team presented their top rated ideas to the steering committee. The steering committee took approximately 7 months to respond to the design team’s proposal; they approved five activities without edit; approved two activities with small changes based on current store logistics; requested more information on four activities; and did not approve four activities (Table 3).

### 3.4. Model Outputs

Of the seven activities that were agreed upon by the steering committee, five were implemented by the design team by the end of the study period; two were not completed because they needed other resources to implement (i.e., waiting on information technology department to complete tasks). Surveys at follow up from 105 store workers (67% response rate) showed the activities noticed most often by workers were ones that were implemented in the breakroom: the new employee refrigerator and discounted bottles of water. Activities that were delivered in other areas of the store were implemented intermittently, and not noticed by many workers. Only six workers said they did not notice any of the implemented program activities. Similarly, utilization of the activities was higher for those implemented in the breakroom and for the activities that did not require much extra effort by the workers. During store worker interviews, workers either were excited about the new health activities and wanted to see more implemented or they had not heard of them. Those that had not heard of the activities indicated that direct communication from store management about new health opportunities may be more useful than printed materials placed throughout the store. Only one of the five workers reported health behavior changes based on an implemented program activity; other workers said that they appreciated the effort, but that none of the implemented activities impacted their personal behaviors.

## 4. Discussion

Implementation of the HWPP was moderately successful in the grocery store setting as demonstrated by good fidelity to program materials, design team engagement in the IDEAS process, and the number of and uptake of program activities in a relatively short time period. This success can be attributed mostly to the design team’s interest in the program and the extra time spent by the facilitator to move the team along; leadership support, including lack of active participation by the store management, was the main barrier to further success. The logic model provided an effective and simple framework for evaluating program implementation and allowed us to better understand the workplace factors necessary for success, as well as challenges or barriers that might be overcome with program modifications or additional resources. The HWPP offers multiple tools that can be used in conjunction with this model including the organizational readiness checklist to evaluate Pre-Implementation and the Management Dashboard and PERS tools to evaluate the Inputs, Activities, and Outputs under Program Implementation.

The program inputs (i.e., HWPP program, design team, steering committee, and facilitator) provided a good structure for the program. The HWPP materials were extremely helpful for the facilitator, although the language was somewhat confusing to the design team. High fidelity to the recruitment criteria led to high engagement and enthusiasm of design team members. The design team’s interest in health and improving their store was vital to their success. The design team members had strong and consistent attendance and participation during meetings, yet seemed to lack the skills needed to progress through all steps of the program. They proceeded well with the initial steps to assess the workplace, identify problems, and come up with solutions, but struggled with the subsequent steps required to create a realistic plan to present to the steering committee. It is likely the design team members had not previously had the need nor opportunity to use these skills in their jobs. Employees may develop these skills through their jobs or by participating in employee-management teams for other business reasons. However, teams consisting of employees without these skills may be unable to effectively design and implement workplace changes without additional external support or training [18,19,54,55].

As a result, the team required substantial assistance from the facilitator to organize information and develop plans to complete each step of the process. The time demands on the facilitator far exceeded our expectations. It is possible that the steering committee or store management could have assisted the design team with some steps. We were careful to include various levels of leadership (including union representation) on the steering committee; however, there was a discrepancy between the stated support (i.e., help with scheduling design team members and help rolling out solutions) and the actual support received (i.e., design team members often not scheduled to work on meeting days and steering committee took little responsibility for implementing activities). Earlier and more frequent involvement from the steering committee in the design team meetings may have mitigated the need for substantial facilitator resources.

The main program activity, the IDEAS Tool, was delivered as intended. With support from the facilitator, the design team was able to meet, agree on a goal, and develop specific activities for each objective to propose to the steering committee (Steps 1–5A). The team’s inability to meet outside of scheduled meetings and the steering committee’s prolonged delay in responding to the design team’s proposal left no time in the study period to complete IDEAS Step 7 (i.e., Evaluation), or initiate another cycle of the IDEAS process. Without this entire action-feedback cycle, the potential for organizational learning was decreased. This long delay also affected morale and enthusiasm, which resulted in two members leaving the design team. Additionally, the design team’s meeting location may have been a problem for some team members. The onsite meeting space was not private; store managers and other employees frequently passed through the meeting space, causing the design team to feel uncomfortable sharing information. Despite these challenges, the program produced worthwhile outputs, demonstrating program success and a positive design team-steering committee collaboration. Overall, the design team had a positive impression of the process noting an increased comradery with team members and healthier behaviors as a result of the intervention. Some team members reported a sense of self-efficacy for continuing the program, while others did not think they could continue without the research team there to facilitate and hold management accountable. Further, data from surveys and interviews showed that store workers were aware of and utilized the workplace activities developed by the design team, indicating relevance to the target audience. Feedback about the methods used for communicating the activities was helpful in explaining possible reasons for non-awareness.

We encountered several challenges during the program that are best described and understood in the pre-implementation and context elements of the logic model. Most importantly, this pilot project grew out of an existing collaboration with a union and three regional grocers. During the planning phases, because only one grocer volunteered to participate and then offered only one store as the test site, we did not have the opportunity to assess organizational readiness at the corporate or the store levels, nor were we able to choose a site that demonstrated readiness to change. While the initial store manager was enthusiastic, he was transferred to another store early in the study and the manager who replaced him was not as invested. The new store manager’s lack of interest in the program filtered down to the design team who felt that their efforts were not appreciated. Over time, the design team’s level of enthusiasm and engagement in the process decreased. Many previous studies have shown that lack of organizational readiness and leadership support are critical factors to program success [18,19,28,55,56,57]. The HWPP program materials describe the importance of organizational readiness but do not provide guidance on how to prevent or remediate diminishing leadership support during the course of implementing the program. In our study, we found that the steering committee and store management were less supportive of interventions that focused on addressing workplace problems (e.g., supply order process and communication) and had fewer concerns about those that focused on changing individual behaviors (e.g., walking program). It is possible that the steering committee did not fully understand the purpose of the program and therefore were less willing to support the design team’s ideas. Assessing organizational and leadership knowledge of the Total Worker Health approach may be an important part of determining program readiness and the need for education or training before and during program implementation.

We also faced obstacles related to the labor-management structure and agreements and differences between the different unions. The design team was challenged to find activities that applied to employees from the various unions, since the health benefits varied between different unions. This made it difficult for the design team to promote or build upon existing health resources. Due to labor contracts, design team members were not allowed to meet on paid work time. The research team addressed this by paying team members for their time to attend meetings; we do not know if the team’s attendance and engagement would have been different had they been allowed to meet on paid work time. Scheduling design team members to work on meeting days also proved to be difficult, which meant that design team members were asked to come in on their days off. These payment and scheduling challenges made some design team members question management’s support and willingness to follow through on proposed activities. The issue of paid time to participate on a design team is a problem when trying to implement a participatory program in hourly-paid workers. Management support should include compensating design team members for their time, and ensuring protected time for team members to develop their ideas.

Research has demonstrated a clear link between worker health and productivity, and investing in employee health has become a popular strategy for improving business outcomes [19,55,56,58]; however, many organizations struggle with supporting worker health initiatives when they compete with business objectives [59]. The design team members in this project recognized the need to fit their ideas into the broader business purpose and were thoughtful in creating activities that capitalized on existing resources or that could be marketed to retail customers in addition to store workers (e.g., premade healthy meals, healthy items near the checkout). While some activities were initially supported by the steering committee, they were not maintained over time because other initiatives, such as holiday product placement, took priority. Additionally, management put little effort into making the existing healthy options for customers more accessible to employees, suggesting that business needs were more important than worker health. This issue of competing interests between business and health is an important contextual factor to consider in interpreting the outcomes of TWH interventions and programs. Other contextual factors that we encountered in this study included seasonality of the work, skill level of employees, rotating employee schedules, and need to put customers first. All of these factors likely influenced the result of the participatory process used in this study, and may impact health and safety initiatives in the retail industry.

Our research study had several limitations. As described, workers were not able to attend meetings during work time and therefore were paid by the research team to attend. The collection of data for the process evaluation may have had an impact on the program’s delivery. It is not known if the successful delivery of the program in one store will be generalizable to other retail locations, with different workers, management, facilities, and culture. In addition, we have limited data on whether the observed program implementation had an effect on the health behaviors of workers.

There were also several strengths to the study, including our relationship with the store that allowed us access to employees and support for the research, in addition to the facilitator’s strong rapport with the design team. The detailed process measures allowed us to evaluate the fidelity of the program implementation and note which components were problematic and should be improved in future trials. The HWPP materials provided a useful structure and guide to make decisions throughout the process.

Participatory methods like those used in the HWPP may be useful in developing TWH interventions that address a variety of work factors that affect worker health. Our recommendations for those who may choose to use this program are: (1) Assess organizational knowledge, readiness, resources, and commitment; build in time prior to implementation to educate leadership and ensure that they understand the program goals, processes, and expectations; (2) Include and budget for a knowledgeable facilitator who has good communication, planning, and organizational skills; (3) Choose team members who are enthusiastic and have good communication, planning, and organizational skills (or ensure that the steering committee can assist); (4) Schedule in-person meeting time to complete the activities for each step (rather than assume the team will complete things outside of meetings); (5) Customize the worksheets for the audience and add materials as necessary to aid the team through the process; (6) Involve the steering committee early in the process and ask them at the onset of the program to play an active role in planning and implementing solutions; and (7) Build in time and resources for periodic evaluation and modifications that may result from the evaluation.

## 5. Conclusions

Participatory programs such as the HWPP show promise as a methodology for creating effective Total Worker Health interventions. This approach is useful for developing activities that can be used by workers and are relevant to their health. This is particularly important for workers in lower paying jobs or in jobs that have complex or chaotic work environments which present other challenges for good health behaviors. The detailed evaluation showed that substantial resources are needed to deliver the program and that enthusiastic, consistent, and active support from management is a critical determinant of success. The broader workplace context may also present challenges which should not be minimized or ignored. Future research studies should explore creative approaches for addressing organizational/contextual challenges that arise during participatory programs and should examine the efficacy of participatory programs. The logic model in this paper offers a framework for evaluating both implementation and efficacy, while considering the unique organizational contexts in which the intervention occurs.

## Figures and Tables

**Table 1:** Demographics of the Baseline Survey Respondents.

	Mean (SD)
Age	42 (15.1)
BMI	28.21 (6.3)
	%
Body Mass Index (BMI) Category	
Underweight	1
Normal weight	37.3
Overweight	28.4
Obese	33.3
Gender	
Female	52.4
Race	
African American	37.5
Caucasian	53.8
Other	8.6
Hispanic/Latino	3
Marital Status	
Married	28.6
Member of unmarried couple	14.3
Never married	41.9
Widowed	4.8
Divorced/separated	10.5
1 or more children live in household	40.4
Highest level of education	
Less than high school	1.9
High school graduate or General Education Diploma	35.6
Postsecondary Education	62.5
Health Behaviors	
Often bring lunch from home	12.5
Eat the recommend amount of fruits	34.2
Eat the recommend amount of vegetables	38.7
Often eat fried foods	17.5
Drink 16oz or more of regular soda or sugary drinks daily	24.2
Eats sweets more than 2 times a day	27.7
Does not meet exercise recommendations	45.2
Considered increasing physical activity	71.2
Health Climate	
Believe organization is fully committed to employee wellness	36.7
Tried to control their weight in the last year	68.3
Stand in place for more than one hour	43.5
No energy to exercise	39.2
No energy for family	33.3
Exhausted after work	61.7

**Table 2:** Participatory Health Program Process Evaluation.

Process Measures & Indicators	Results
Inputs	
Fidelity to HWPP materials	
Used IDEAS Tool materials/worksheets as planned	Yes—minor language modifications
Design team members understood the materials/program process	Yes—design team members reported that materials were easy to understand, but didn’t always know the best way to move forward through program materials
Facilitator	
Knowledgeable about the HWPP & IDEAS Tool	Yes—thorough review of facilitator guide prior to program initiation
Knowledgeable about the workplace	Partial—external researcher with previous experience in this store
Time expenditure met expectations (~20 h)	No—greater than anticipated (57 h over 10 weeks)
Design Team	
Recruited 6–8 design team members	Yes—6 design team members
Met recruitment criteria	Yes—met all criteria
Design team members scheduled to work on meeting days	No—all design team members scheduled to work on only 2 of 9 meeting days
Steering Committee	
Steering committee represented various levels of authority	Partial—corporate, store supervisor, unions; store manager not involved
Activities	
Fidelity to the IDEAS Tool	
Design team completed IDEAS Steps 1–5A	Yes—completed Steps 1–5A; also partially completed Step 6
Steering committee completed IDEAS Steps 5B–6	Partiall—completed Step 5B; partially completed Step 6
Dose	
Number/duration/frequency of design team meetings	16 meetings; 50–60 min each; met weekly for 10 weeks, then as needed
Number/duration/frequency of steering committee meetings	2 meetings; 60–90 min each; 7 months between meetings
Engagement	
Design team meeting attendance	All present at six of 16 scheduled meetings; one member absent at seven meetings; two or more members absent at three meetings
Steering committee meeting attendance	All present at 1 of 2 scheduled meetings; 2 members present at second meeting
Design team engagement (Facilitator mean rating for each design team members across all meetings; Scale: 0 = No, 0.5 = some/somewhat, 1 = Yes)	Offered new ideas during meetings = 0.86
	Actively participated in meeting = 0.88
	Completed homework = 0.50
	Discussed projects with co-workers = 0.81
	Design team required significant facilitation to further develop and implement activities; facilitator took on a lot of activity development responsibility; team members reported they were not motivated to take initiative, however they often made a point to attend team meetings even when not scheduled to work (15 out of 20 instances)
Design team perception of the process	Team members reported feeling positively impacted by the program and thought the program was innovative and important, but they did not know how to implement activities without help.
Design team perception of support	The team did not feel they received logistical support from store management to implement solutions and response time was slow. They also felt that the steering committee did not follow through on promises and took too long to respond to the team.
Steering committee perception of program	1 of 6 steering committee members continued with the program until completion; one member was vocal about not believing in the program/process.
Activities generated	The design team generated 3 objectives with 15 distinct activities; the steering committee approved 7 activities
Outputs	
Store Worker Reach	
Activities implemented	5 activities were implemented
Awareness of implemented solutions	Surveys: 99 of 105 workers noticed at least one activity implemented by the design team. Awareness varied by activities; Results shown in Table 3.
Utilization of implemented activities	Surveys: Participation in the activities was higher among workers who used the break room, where most of the activities were implemented and communicated to the workforce. Results shown in Table 3.
Store Workers’ Perception of Program	Surveys: 39 of 105 workers reported the activities helped them improve their eating and/or exercise habits
	Worker interviews (*n* = 5): 4/5 thought the activities were good for store workers in general, but changes in their own health behaviors were made for other reasons, not due to program

Note: HWPP: Healthy Workplace Participatory Program, IDEAS: Intervention Design and Analysis Scorecard.

**Table 3:** Proposed activities and implementation outcomes.

Objectives and Activities	Steering Committee Response to Proposal	Implemented (Yes/No)—Responsible Party	Store Workers	
			Noticed (*n* = 105)	Used (*n* = 105)
Improve Store Communication				
Utilize email to communicate info	Agreed	No—store mgmt.	-	-
Use TV in break room for announcements	Agreed with modifications	No—store mgmt.	-	-
Develop better process for tracking and ordering supplies (identified as a stressor)	Not approved (said it was not relevant to the project)	N/A	-	-
Improve Diet at Work				
Get a bigger refrigerator for break room	Agreed	Yes—store mgmt.	78%	43%
Healthier options near checkout	Agreed	Yes (partial)—store mgmt.	30%	16%
Bottled water in break room	Agreed	Yes—design team	81%	47%
“Healthy choices” section	Wanted more details	No—design team	-	-
Include healthy options in $5 meals	Wanted more details	No—steering committee	-	-
Offer healthier premade meals and offer discount	Not approved (not profitable)	N/A	-	-
Add nutrition info and healthy recipes to recipe kiosks	Not approved (kiosks no longer used)	N/A	-	-
Reward workers for eating healthy	Wanted more details	No—design team	-	-
Increase Health Awareness				
Walking challenge with incentives	Agreed	Yes (Completed one 12-week challenge) —design team	50%	13%
Health focused newsletter	Agreed	Yes (2 delivered during study period)—design team	45%	25%
Gym/ Exercise class discounts	Need details from unions	No—steering committee	-	-
Add more health topics to the “Meet the Expert” class schedule & increase the number of classes	Not approved (no longer offer classes)	N/A	-	-
